# Immune niche-associated and niche-regulated plasticity in glioblastoma: state transitions, immune escape, and therapeutic vulnerabilities

**DOI:** 10.3389/fimmu.2026.1857659

**Published:** 2026-06-26

**Authors:** Haijun Zhang, Xiaofang Wu, Haojie Liao, Yifan Zhang

**Affiliations:** Department of Neurology, Shenzhen Baoan People’s Hospital, Shenzhen, China

**Keywords:** cell plasticity, glioblastoma, glioma, immune escape, microglia, therapeutic vulnerabilities, tumor immune niche, tumor-associated macrophages

## Abstract

Glioblastoma (GBM), the most aggressive diffuse glioma in adults, remains one of the most lethal malignancies of the central nervous system owing to its profound heterogeneity, near-universal recurrence, and marked resistance to therapy. Increasing evidence indicates that these features cannot be fully explained by genetic alterations alone but instead reflect a high degree of cancer cell plasticity shaped by dynamic interactions with the tumor microenvironment. In this review, we discuss how the tumor immune niche is associated with, and in selected experimental settings may influence, GBM cell-state transitions. Single-cell and spatial studies support the view that GBM cells may shift among NPC-like, OPC-like, AC-like, mesenchymal-like, stem-like, and stress-adapted states under immune, hypoxic, and therapeutic pressures. Among these influences, tumor-associated macrophages, microglia, inflammatory cytokines, and hypoxic or perivascular niches have been implicated in the enrichment of aggressive phenotypes and the maintenance of immunosuppressive ecosystems. We further highlight how plasticity-driven state transitions contribute to immune escape by enhancing myeloid recruitment, limiting effective T-cell function, and reinforcing mesenchymal and injury-response programs. Therapy adds another layer of complexity by inducing adaptive reprogramming in surviving cells, thereby fostering recurrence-associated states with increased resistance to treatment. Finally, we propose that durable GBM control will likely require combinatorial strategies that target both tumor cell plasticity and the immune niche that sustains it. Understanding this reciprocal relationship may reveal new therapeutic vulnerabilities and improve the design of future immunomodulatory and anti-plasticity interventions.

## 1 Introduction

Diffuse gliomas are among the most lethal primary tumors of the central nervous system, with glioblastoma, IDH-wildtype, representing the most aggressive adult-type diffuse glioma despite multimodal treatment including maximal surgical resection, radiotherapy, and temozolomide-based chemotherapy (1, 2). Although immunotherapy has transformed the management of several peripheral malignancies, comparable clinical benefit has not been achieved in GBM. In the first randomized phase 3 trial of PD-1 blockade in recurrent glioblastoma (CheckMate 143), nivolumab failed to improve overall survival relative to bevacizumab, and checkpoint-inhibitor studies and clinical reviews have shown limited benefit in unselected GBM populations (3, 4). These clinical results highlight the profoundly immunosuppressive and therapy-resistant nature of the GBM tumor microenvironment (TME). This resistance is increasingly attributed in part to limited and frequently dysfunctional lymphocyte infiltration (5, 6). It is also linked to the dominant presence of immunoregulatory myeloid populations within the tumor milieu (7).

Indeed, GBM tumors are characterized by extensive accumulation of glioma-associated myeloid cells, primarily composed of resident microglia and bone marrow-derived macrophages, which together can constitute a substantial fraction of the tumor cellular compartment, in some studies approaching half of the tumor mass (7, 8). Rather than functioning as passive bystanders, these populations have been implicated in tumor-supportive processes; primary single-cell and functional studies specifically support immunomodulatory myeloid programs, myeloid-linked MES-like state changes, and inflammatory myeloid recruitment (9). Broader functions such as angiogenesis and extracellular-matrix remodeling remain context-dependent and have been summarized in prior reviews (10). Importantly, emerging evidence indicates that these myeloid populations are highly heterogeneous in both ontogeny and function, suggesting specialized roles within distinct tumor regions (7).

Recent advances in single-cell and spatially resolved transcriptomic technologies have further shown, and recent reviews have synthesized, evidence that glioma-associated myeloid cells are not uniformly distributed throughout the TME but instead segregate into anatomically restricted microdomains shaped by local metabolic, vascular, and stromal cues (7, 11). These observations challenge traditional bulk descriptions of the TME and support an evolving framework in which tumor-infiltrating immune cells can form spatially organized immunological niches that coordinate context-dependent interactions between malignant, immune, and stromal compartments (11, 12). In GBM, the establishment of such niches is further influenced by the specialized immune architecture of the central nervous system, including resident myeloid populations, brain-border immune niches, meningeal lymphatic drainage, and neuroimmune signaling pathways (13, 14). This mini-review is GBM-centered rather than a broad survey of all gliomas, focusing on primary single-cell and functional studies that link malignant cell-state plasticity to immune niche architecture (9, 15). Integrative subtype analyses are considered where they connect cell-state programs with immune remodeling (16). Its distinction from prior reviews of GBM plasticity, tumor-associated macrophages/microglia, or immunotherapy resistance is that it organizes these data around reciprocal tumor-immune niche interactions and the strength of evidence supporting each link. We first define the cell-state framework used in recent single-cell and spatial studies, then examine the key primary studies connecting malignant states with local immune architecture—distinguishing correlative associations from perturbation-based causal evidence and emphasizing myeloid-rich, hypoxic, perivascular, and therapy-altered contexts in which MES-like, stem-like, and stress-adapted transitions have been observed or tested (9, 11). Finally, we examine how these states reinforce immune escape and resistance and highlight investigational vulnerabilities arising from targeting both malignant-cell plasticity and the immune niche that sustains it.

## 2 Immune niche-dependent state transitions in glioblastoma

Throughout this review we use the cell-state terminology of single-cell glioblastoma studies: NPC-like (neural progenitor-like), OPC-like (oligodendrocyte progenitor-like), AC-like (astrocyte-like), and MES-like (mesenchymal-like) programs; stem-like for tumor-propagating or self-renewal features that overlap with but are not identical to these states; and stress-adapted for injury-, hypoxia-, therapy-, or inflammatory-response programs that may emerge across state boundaries. These are transcriptional and functional states rather than fixed lineages.

These findings complicate the traditional view of tumor heterogeneity. In GBM, tumor-cell populations are not always independent clonal branches evolving in parallel; cells often occupy a looser state, switching between or resting in partially stable configurations. This flexibility lets them change phenotype in response to microenvironmental and transcriptional cues (17, 18). Treatment can further reshape these cellular programs during tumor evolution (19), and stemness in glioblastoma accordingly behaves not as a fixed property of a small pre-existing population but as a reversible, microenvironment-induced state (20). However, these transformations do not occur randomly. They are constrained by developmental programs, tumor genetic background, and microenvironmental conditions, and pathway-defined and metabolic subtypes have been linked to these state preferences (21). In this context the immune niche is increasingly viewed not as a vague background signal but as a potential regulator of which tumor-cell states are occupied and when switching occurs. The GBM immune compartment is dominated by resident microglia and infiltrating macrophages that comprise distinct programs differing in origin, spatial distribution, and immunomodulatory function (7). Where perturbation data exist, these myeloid populations appear to influence which malignant phenotypes are selected or maintained (9). Tumor areas with strong inflammatory activity and myeloid infiltration often accumulate mesenchymal-like tumor states (16), and functional studies suggest macrophage-derived signals can drive GBM cells toward a more invasive, stress-adapted, and less therapy-sensitive phenotype (9). Several original studies have supported a bidirectional interaction model, although this relationship may only be most clear in specific biological contexts. The mesenchymal tumor program is associated with increased infiltration of myeloid cells (16); macrophage-derived OSM can promote GBM cells to acquire a MES-like state (9). Conversely, GBM-derived IL-33 can recruit and activate myeloid cells, thereby maintaining an inflammatory microenvironment conducive to tumor progression (22). This does not mean that every region enriched with myeloid cells will drive the same tumor cell state. A more cautious understanding is that only when appropriate spatial and molecular conditions coexist do malignant cell programs and immune programs mutually reinforce each other. Hypoxia is one of the strongest local factors among these. Hypoxic and perivascular regions have been associated with stemness, metabolic adaptation, mesenchymal-like programs, and changes in immune-cell recruitment (23, 24), but whether these niches truly induce state transitions and how important this role is still requires functional experimental verification.

**Figure 1:**
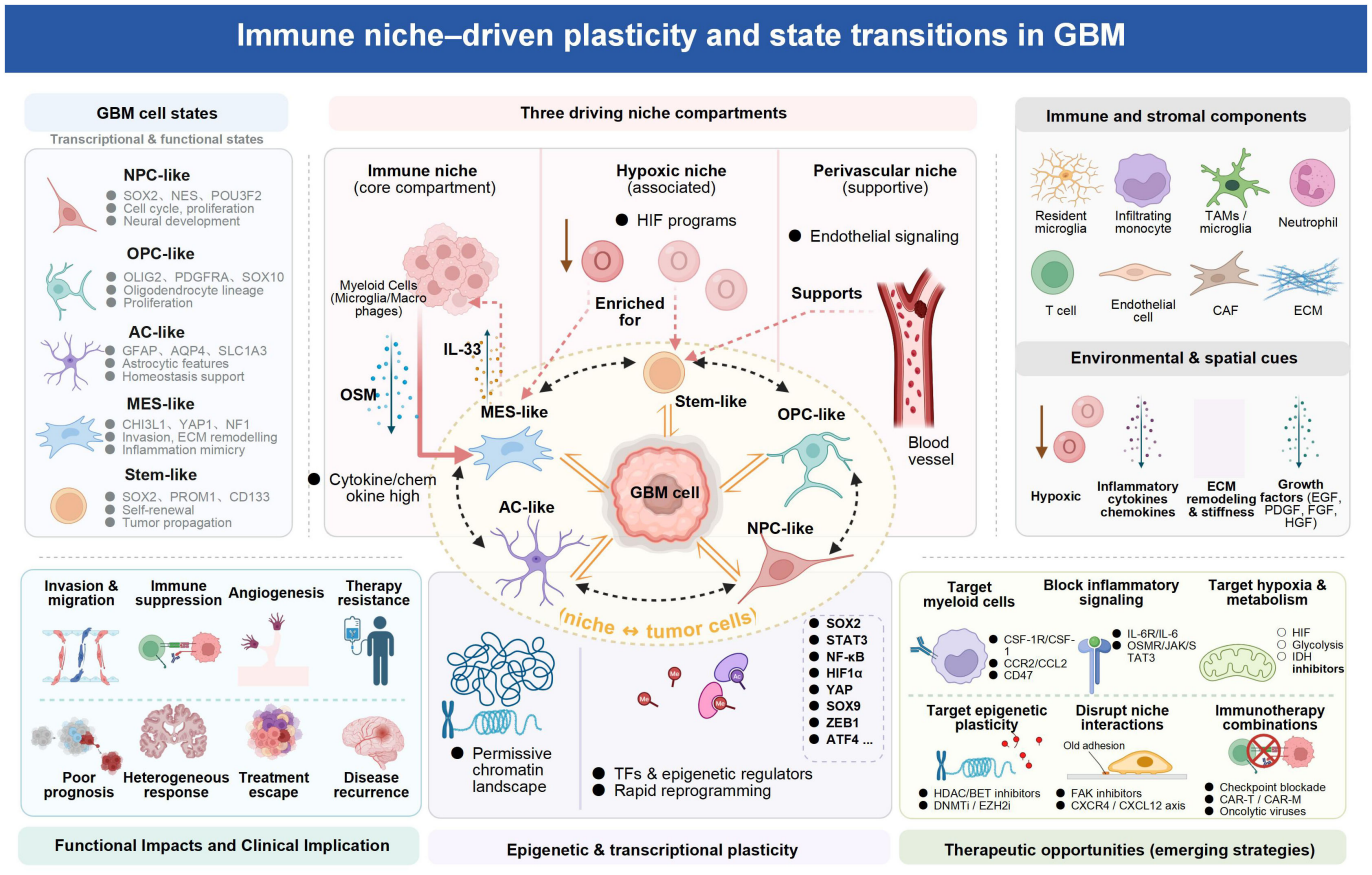
Immune niche-driven plasticity and state transitions in glioblastoma(GBM). The schematic summarizes how distinct immune, hypoxic, and perivascular niches shape malignant cell-state plasticity in glioblastoma (GBM). GBM cells can occupy multiple transcriptional and functional states, including NPC-like, OPC-like, AC-like, MES-like, and stem-like programs. Within the immune niche, myeloid cells, including resident microglia and infiltrating macrophages, may promote MES-like transition through inflammatory cytokines and chemokines, including OSM- and IL-33-related signaling. Hypoxic regions enrich HIF-associated programs and support stem-like and MES-like adaptation, whereas perivascular niches provide endothelial and stromal signals that help maintain stem-like and OPC-like states. These niche-derived cues interact with permissive chromatin states, transcription factors, and epigenetic regulators to facilitate rapid reprogramming among malignant cell states. Functionally, this plasticity contributes to invasion, immune suppression, angiogenesis, treatment escape, heterogeneous therapeutic response, poor prognosis, and disease recurrence. The lower right panel highlights emerging therapeutic opportunities, including targeting myeloid cells, blocking inflammatory signaling, disrupting niche interactions, inhibiting epigenetic plasticity, targeting hypoxia and metabolic adaptation, and developing immunotherapy-based combination strategies.

In hypoxic areas, GBM cells often upregulate programs for survival, migration, angiogenesis, and developmental plasticity, while myeloid cells entering these regions acquire immunosuppressive phenotypes that may further promote malignant adaptation (23, 24). Perivascular regions appear to act similarly: proximity to endothelial cells and extracellular matrix can create conditions that help tumor cells maintain a stem-like state (12, 25). Underlying these phenomena is epigenetic and transcriptional plasticity—GBM cells often retain a relatively open chromatin state, so that inflammatory, hypoxic, metabolic, or therapy-induced cues can rapidly reshape lineage programs across state boundaries (25, 26). Transcription factors and epigenetic regulators linked to neural development, mesenchymal activation, and injury response may therefore not merely mark identity but actively drive adaptation, particularly where immune and stromal signals converge to push developmental-like reprogramming (27). Altogether, current evidence supports a model in which GBM state transitions are not only cell-intrinsic phenomena but also ecological responses associated with immune and spatial niches, and in selected settings influenced by them. The local abundance, composition, and activation state of myeloid cells; the degree of hypoxia and vascular dysfunction; and the accessibility of epigenetic regulatory circuits together shape whether GBM cells remain in relatively differentiated states or shift toward mesenchymal, stem-like, or stress-adapted identities. When supported by functional evidence, this framework helps explain how cellular plasticity becomes linked to invasion, recurrence, and treatment resistance (**Figure 1**).

Several primary studies are central to the current niche-plasticity framework. Neftel et al. used single-cell RNA sequencing of human glioblastoma to define recurrent NPC-like, OPC-like, AC-like, and MES-like malignant cell states, building on earlier single-cell evidence of intratumoral heterogeneity in primary glioblastoma, and showed that individual tumors contain mixtures of these states rather than a single fixed subtype (15, 28). Their analysis further links the composition of cellular states with genetic alterations and microenvironmental context, providing a basis for understanding GBM heterogeneity as a dynamic cellular state system, rather than just as parallel clonal diversity (15). Wang et al. linked this cellular state view to the immune microenvironment, showing that the glioma-intrinsic expression subtypes evolve with immune changes, among which the mesenchymal-like program is associated with increased infiltration of myeloid cells and enhanced inflammatory signaling (16).

Functional evidence regarding causal relationships is relatively limited, but particularly important. Hara et al. demonstrated that the interaction between cancer cells and immune cells can drive MES-like transitions and identified the macrophage-derived oncostatin M and OSMR/LIFR-GP130-STAT3 signaling pathway as a mechanism that promotes the acquisition of mesenchymal-like states (9). This study provides one of the clearest pieces of perturbation-based evidence linking myeloid-derived signals with malignant cell-state transition. In contrast, many other single-cell or spatial studies primarily support co-localization relationships or inferred ligand-receptor interactions rather than direct causal relationships (9, 11). These differences are important because unless supported by functional validation, myeloid cell enrichment, hypoxia, or perivascular localization should not be automatically interpreted as having a direct inducing effect.

Recent work has extended this framework across tissue scale and treatment history. Wang et al. built a single-cell atlas of glioblastoma evolution under therapy and identified treatment-associated malignant and microenvironmental programs that may serve as cell-intrinsic and cell-extrinsic targets (19). Mathur et al. used a 3D whole-tumor atlas to show that GBM heterogeneity is spatially organized across tumor regions, tying local tissue structure to malignant-state composition (29). Miller et al. dissected the programs, origins, and immunoregulatory roles of glioma myeloid cells, showing that resident microglia and infiltrating macrophages are not interchangeable but differ by region and function (7). Together, these primary studies move the field from a descriptive catalogue of states toward a more testable, niche-centered model of glioblastoma plasticity.

Interpreting this literature requires an evidence hierarchy. Direct perturbation studies, such as macrophage-derived OSM driving MES-like transition or glioma-derived IL-33 recruiting inflammatory myeloid cells, provide stronger causal support for tumor-immune niche regulation (9, 22). Single-cell, 3D-mapping, and spatial studies define co-occurring malignant states, myeloid programs, and anatomical niches at high resolution but remain primarily associative unless paired with perturbation or longitudinal validation (11, 29). Spatial enrichment of MES-like, stem-like, or stress-adapted states in myeloid-rich, hypoxic, or perivascular regions should therefore be read as niche association, and stronger causal language reserved for experimentally validated interactions.

## 3 Plasticity-mediated immune escape in glioblastoma

### 3.1 Plastic state transitions reshape an immunosuppressive microenvironment

One of the most clinically significant consequences of GBM plasticity is its tight coupling to immune escape. Immune suppression in GBM is not simply a background condition imposed by the brain or tumor mass; it can be actively reinforced by tumor cell-state changes that alter how malignant cells communicate with surrounding immune populations (16). Specific reciprocal tumor-myeloid mechanisms have been supported by perturbation studies (9, 22). As GBM cells transition toward mesenchymal-like or stress-adapted phenotypes, primary studies show enrichment of inflammatory signaling, extracellular-matrix or wound-response features, and myeloid-associated immune remodeling (16). NF-κB-linked mesenchymal differentiation has also been tied to radioresistance (30). Mesenchymal-like transition can be driven directly by myeloid-derived signals (9). It is accompanied by immunological changes in the microenvironment (16). NF-κB-dependent mesenchymal differentiation programs can reinforce this phenotype (30). In parallel, GBM-derived mediators such as IL-33 can recruit and activate myeloid cells, thereby sustaining an inflammatory tumor-promoting microenvironment (22). These programs are generally not associated with productive antitumor immunity in GBM; instead they establish an immune landscape dominated by dysfunctional myeloid cells, reduced effector lymphocyte activity, and persistent inflammatory signaling that favors tumor persistence. Tumor-associated macrophages, infiltrating monocytes, and resident microglia are central to this process, with primary studies showing myeloid programs linked to immunomodulatory function and malignant-state remodeling (7, 9). More specifically, Hara et al. identified macrophage-derived OSM as a driver of MES-like transition (9), while De Boeck et al. showed that glioma-derived IL-33 can orchestrate inflammatory myeloid recruitment and accelerate glioma progression (22). This reciprocal loop may explain why mesenchymal transition in GBM is so often seen together with immune-rich but functionally suppressive tumor regions, although this link should be interpreted most confidently in settings where perturbation data support it (9, 16). Mesenchymal-like GBM cells are central here, frequently upregulating wound-response, inflammatory, and myeloid-recruitment genes, which positions the mesenchymal state as a principal interface between malignant plasticity and immune suppression (16, 30). Cytokine and chemokine signaling are involved in this process. Specific cytokine axes, including OSM-OSMR/LIFR-GP130-STAT3 and IL-33-driven activation of myeloid cells, link inflammatory niches with malignant cell-state transition and myeloid-cell recruitment (9, 22). The broader impact of these signals on T-cell dysfunction remains context-dependent and should be interpreted alongside evidence of T-cell exhaustion and sequestration in GBM (5, 6). In this way, GBM plasticity may shift the immune microenvironment away from effective immune surveillance and toward conditions more favorable for tumor cell survival, invasion, and immune escape.

### 3.2 Immune dysfunction, lymphocyte exclusion, and the self-reinforcing loop of plasticity

The immunosuppressive niche formed by plastic GBM states is further reinforced by severe adaptive-immune dysfunction. T cells, though present in many GBM tumors, are typically sparse, spatially excluded, metabolically constrained, or functionally exhausted, so effective antitumor responses remain limited even when tumor antigens are recognizable (5, 6). Multiple mechanisms can explain this phenomenon, including impaired antigen presentation, upregulation of inhibitory ligand expression, exposure to immunosuppressive cytokines, abnormal antigen processing in myeloid cells, and metabolic conditions that are unfavorable for sustained effector T-cell activity (5, 31). However, these features should not be seen as processes that operate independently of the malignant-cell state. As GBM cells shift toward mesenchymal-like, stem-like, or injury-response-related programs, they may become more difficult for the immune system to recognize or less susceptible to immune attack (16, 30). At the same time, they may also gain stronger abilities to attract immunosuppressive myeloid-cell populations and restrict the entry or sustained presence of functional T cells (6, 16). In this sense immune escape is not only imposed by the external microenvironment but is partly a product of the tumor’s own phenotypic evolution—a self-reinforcing circuit. Tumor-state plasticity can promote immune suppression through myeloid recruitment and inflammatory signaling (9, 22). Subtype-related immune differences also point to a close link between malignant-cell state and myeloid dominance (16). T-cell dysfunction and sequestration further weaken adaptive immunity in GBM (5, 6). This may explain why some GBM tumors resist checkpoint blockade despite detectable immune infiltration: the infiltrating cells are absorbed into a tumor-supportive niche rather than organized into effective antitumor responses. Work on neuron-GBM communication and neural-immune-tumor interactions adds a further layer, since neuronal activity and neuron-derived signals can directly support GBM growth and plasticity and may intersect with immune signaling in the same ecosystem (13, 32). Spatial studies point the same way: the immunosuppressive niche is unevenly distributed, clustering in regions marked by necrosis, vascular failure, local myeloid aggregation, and specific malignant states (11, 29). Immune escape in GBM is thus spatially organized and tightly coupled to tumor-cell state—GBM cells progressively adopt, and may even help build, phenotypes suited to immunosuppressive environments. Countering immune escape may therefore require not only restoring lymphocyte function but also blocking or reversing the state transitions that establish inhibitory niches.

## 4 Therapy-induced plasticity and therapeutic vulnerabilities

A core challenge in GBM therapy is that treatment does not merely clear sensitive tumor cells but also reshapes the environment on which residual cells depend, so recurrence cannot be attributed solely to expansion of drug-resistant clones. Part of the reason is treatment-induced plasticity: surviving cells shift toward more stem-like, mesenchymal, invasive, or stress-tolerant states (19, 29). Single-cell and longitudinal studies show that post-treatment GBM often acquires recurrence-associated programs enriched for damage responses, stromal-like features, and adaptation to immunosuppressive niches (19, 33). Treatment history is thus not merely the backdrop to recurrence but an active force shaping tumor evolution.

Radiotherapy and temozolomide sharpen this point. Both impose DNA-damage and stress-response pressure on surviving cells, with effects that are not purely cytotoxic: radiotherapy has been tied to NF-κB-dependent mesenchymal differentiation and radioresistance (30), while temozolomide-associated hypermutation has been reviewed as an evolutionary consequence of treatment (34). Larger genomic analyses link post-treatment hypermutation to mismatch-repair deficiency (35), and recurrent glioma evolution has been traced in longitudinal mutational studies (36). Notably, this post-treatment hypermutation usually traces to mismatch-repair defects and does not reliably confer responsiveness to PD-1 blockade (35). Increased molecular diversity and antigenic load therefore need not yield immune benefit when the local niche remains dominated by dysfunctional myeloid cells, weak antigen presentation, and T-cell exclusion—the clinical significance of any treatment-induced change depends on the microenvironment in which it occurs.

Anti-angiogenic therapy poses a similar paradox: it can transiently relieve edema and disrupt tumor vasculature, but evasive resistance mechanisms are well recognized (37). Experimental studies indicate that anti-VEGF treatment can aggravate hypoxia or tissue stress and push tumor cells toward more invasive, stromal-like phenotypes (38, 39). Immunotherapy resistance, in turn, likely reflects a combination of antigen-presentation defects, immunosuppressive cytokine networks, and the capacity of specific malignant-cell states to coexist with myeloid-rich niches. Primary GBM datasets point to T-cell dysfunction and sequestration (5, 6). Myeloid-dominated immune states are supported by recent single-cell analyses (7). These observations suggest that therapeutic resistance in GBM often reflects adaptive reprogramming rather than simple pathway escape.

These observations also suggest testable therapeutic hypotheses. If recurrent glioblastoma depends partly on niche-supported plasticity, the pathways enabling such adaptation may represent context-dependent vulnerabilities rather than validated targets. Myeloid-directed strategies, including CSF1R, CCL2/CCR2, STAT3, and TGF-β-related approaches, have been proposed to weaken cues that stabilize immunosuppressive and MES-like states (40, 41). Metabolic strategies remain investigational in glioblastoma (42). Epigenetic strategies may restrict lineage switching and adaptive persistence, but their clinical utility is still unproven (43). A rational but still investigational approach is therefore a combinatorial strategy: standard therapy to reduce tumor burden paired with niche-remodeling or anti-plasticity interventions selected by measurable tumor-cell states, immune composition, and treatment history, framed as hypotheses for biomarker-guided trials rather than established strategies (**Table 1**).

**Table 1:** Therapy-induced plasticity and therapeutic vulnerabilities in GBM.

Therapy context	Plasticity-associated changes	Immune/microenvironmental impact	Therapeutic implication	Key supporting literature
Radiotherapy and temozolomide	Induction of stem-like, mesenchymal, invasive, and stress-adapted states	DNA damage, oxidative stress, and inflammatory signaling reshape the niche and favor recurrence (30, 35)	Combining standard therapy with anti-plasticity approaches is a candidate strategy requiring validation	(19, 30)
Temozolomide-associated hypermutation	Increased molecular diversity without consistent therapeutic benefit	Immunogenic potential may be offset by myeloid dominance, poor antigen presentation, and T-cell exclusion	Hypermutation alone may be insufficient; niche remodeling may be needed	(34, 35)
Anti-angiogenic therapy	Selection of invasive and mesenchymal phenotypes	Aggravates hypoxia and tissue stress, reinforcing an adaptation-permissive niche (38, 39)	Could be tested in combination with anti-hypoxia or immune-modulating strategies	(38, 39)
Immunotherapy	Adaptive immune evasion through altered antigenicity and state transition	Persistent immunosuppressive microenvironment limits durable responses	Combination regimens targeting both immune escape and plasticity are a testable hypothesis	(5, 31)
Myeloid-targeted strategies	Reduction of niche signals supporting adaptive states	May weaken macrophage/microglia-driven stabilization of mesenchymal and immunosuppressive phenotypes	CSF1R, CCL2/CCR2, STAT3, and TGF-β axes are candidate targets	(41, 44)
Metabolic targeting	Disruption of adaptive survival programs under treatment stress	May have the potential to improve both tumor control and immune function	Exploiting metabolic dependencies may enhance treatment response	(42)
Epigenetic targeting	Restriction of lineage switching and adaptive persistence	May limit transcriptional reprogramming associated with resistance	Whether epigenetic therapy can limit recurrence-associated state transitions requires validation	(43)
Rational combination therapy	Simultaneous suppression of tumor burden and adaptive evolution	Is intended to counteract both malignant reprogramming and supportive niche remodeling	Candidate strategy for durable GBM control, pending clinical validation	(45, 46)

## 5 Discussion and future perspectives

Overall, GBM progression is increasingly understood as a dynamic interaction between plastic malignant cells and immune or spatial niches that are associated with, and in selected experimental settings can influence, their behavior (47, 48). This perspective integrates several recurring features of GBM biology—mesenchymal enrichment at recurrence, persistent stem-like populations, myeloid dominance, and the limited durability of current immunotherapy—and reframes immune escape not as a simple immunological failure but as a product of co-evolution between tumor-cell states and microenvironmental states that gradually reinforce each other (49).

Several limitations should be noted. Most human single-cell and spatial datasets remain observational and cannot, on their own, establish whether immune composition drives malignant states or malignant states reshape immune composition. GBM samples are also shaped by factors that are hard to control—corticosteroid exposure, surgery, radiotherapy, temozolomide, anti-angiogenic therapy, and regional sampling bias—each of which can alter both immune composition and transcriptional state. Biological scope is a further caveat: mechanisms defined in IDH-wildtype GBM should not be assumed to apply verbatim to IDH-mutant or pediatric gliomas. Finally, vulnerabilities inferred from cell-state or niche analysis remain hypotheses until functionally validated and, where appropriate, tested in patient-stratified trials.

Important uncertainties remain. It is still unclear which immune-derived signals cause only transient adaptation and which drive stable reprogramming of GBM identity (50). Primary and recurrent tumors may follow different ecological rules, since recurrent GBM tumors carry a treatment-shaped microenvironment distinct from diagnosis (33). Translationally, single-cell and spatial studies have improved descriptive resolution, but more functional work is needed to establish which interactions are causal and therapeutically actionable (11, 33). Future progress will likely depend on stratifying patients by cell state, spatial niche organization, and treatment history rather than histology alone (11).

From a therapeutic standpoint, durable control of GBM may require more than clearing tumor cells: it may also mean cutting off the niche signals that support adaptive change and limiting the capacity of malignant cells to enter stress-tolerant, immune-escape states (45). Longitudinal lineage analysis, spatial multi-omics, and well-designed combination therapy should therefore be central to the next stage of GBM research and treatment design.
